# Analytical Methodologies for the Characterization and Analysis of the Parent Compound and Phase I Metabolites of 4F-MDMB-BICA in Human Microsome, Urine and Blood Samples

**DOI:** 10.1093/jat/bkab004

**Published:** 2021-01-06

**Authors:** Tímea Körmöczi, Éva Sija, László Institóris, Éva M Kereszty, István Ilisz, Róbert Berkecz

**Affiliations:** Institute of Pharmaceutical Analysis, Faculty of Pharmacy, University of Szeged, Somogyi utca 4, Szeged 6720, Hungary; Department of Forensic Medicine, Faculty of Medicine, University of Szeged, Kossuth Lajos sgt. 40, Szeged 6724, Hungary; Department of Forensic Medicine, Faculty of Medicine, University of Szeged, Kossuth Lajos sgt. 40, Szeged 6724, Hungary; Department of Forensic Medicine, Faculty of Medicine, University of Szeged, Kossuth Lajos sgt. 40, Szeged 6724, Hungary; Institute of Pharmaceutical Analysis, Faculty of Pharmacy, University of Szeged, Somogyi utca 4, Szeged 6720, Hungary; Institute of Pharmaceutical Analysis, Faculty of Pharmacy, University of Szeged, Somogyi utca 4, Szeged 6720, Hungary

## Abstract

4F-MDMB-BICA is one of the most dangerous new illicit synthetic cannabinoids (SCs) in 2020. Consumption of 4F-MDMB-BICA has been associated with a number of death cases and related serious adverse health effects in Hungary. Therefore, the use of reliable analytical methods to confirm the intake of 4F-MDMB-BICA is an important issue in forensic practice. Besides the detection of the parent compounds of SCs, the screening of their metabolites provides a reliable confirmation of their consumption, in particular, when the parent compound is under the limit of detection. To the best of our knowledge, this is the first report describing the identification of metabolites of 4F-MDMD-BICA after treatment with pooled human liver microsome (pHLM) and in human urine and blood samples using the combination of data obtained by comprehensive ultra-high performance liquid chromatography coupled to quadrupole-Orbitrap high-resolution mass spectrometry (UHPLC–HRMS) and semi-targeted UHPLC–HRMS-MS methods. Finally, our routine UHPLC coupled with triple-quadrupole tandem low-resolution mass spectrometry method for screening urine and blood SCs was improved by adding the parent compound and selected main biomarkers of 4F-MDMD-BICA. From the pHLM assay of 4F-MDMD-BICA, 30 phase I metabolites were characterized and structural information thus obtained provided the basis of further identification of *in vivo* urine and blood metabolites. Overall, 20 urinary and 13 blood *in vivo* metabolites of 4F-MDMD-BICA have been identified by the investigation of five authentic urine and two blood samples. The ester hydrolysis metabolite was selected as a reliable primary biomarker in urine and blood. As secondary targets, urinary mono-hydroxylation metabolite and ester hydrolysis + dehydrogenation metabolite in blood were recommended due to their abundance and selectivity. Overall, the main phase I metabolites of 4F-MDMD-BICA were successfully characterized, and our routine analytical method with related sample preparation procedure provided a reliable analytical tool for screening both 4F-MDMD-BICA and its selected metabolites in urine and blood samples.

## Introduction

Synthetic cannabinoids (SCs), as psychoactive substances, have been one of the main representatives of illegal drugs in recent years. According to the seizure data of the last few years in Hungary, SCs are the most frequent designer drugs (1, 2). The SCs have well-defined core structures and a wide variety of combination of the different tails, linkers and linked groups. Their effects are similar to those of tetrahydrocannabinol (THC), the main psychoactive component of cannabis, as they are strong agonists of the cannabis receptors CB1 and CB2 (3, 4). The easy availability and low cost of SCs compared to those of traditional drugs have resulted in widespread consumption of SCs among teens, drug users, prison population and low-income people (1, 2). The numerous severe intoxications and several fatal cases of SCs can be attributed to the lack of information of dosage in the period of introduction, the unpredictable and adverse effects of the new substances and the lack of specific diagnostic and therapeutic measures at the emergency care (5–9). Thus, the confirmation of the use of SCs from body fluids is a critical issue in emergency, toxicology and forensic practices. In several cases, despite severe intoxication symptoms, the concentration of SC was very low in urine and blood (10, 11). Consequently, sensitive analytical methods are important for reliable quantification of the parent compounds of the given SCs. The metabolomics approach in the analysis of SCs in biological samples is also appropriate to confirm the abuse of this kind of psychoactive substances. The analysis of the main metabolites of SCs is expedient in routine analysis owing to their rapid metabolism resulting in decreasing concentration of the parent compound in urine and blood within a relatively short duration. Thus, the detection window for the SC use can be extended. However, special caution should be exercised in selecting appropriate metabolites. This is to avoid falsely identifying a substance due to mistaking detected metabolites for those of other known SCs (5, 12). In general, the selection of at least two main metabolites is recommended in routine analysis to obtain reliable qualitative data to prove SCs consumption. *In vitro* assays using pooled human liver microsome (pHLM) for the production of metabolites of SCs are frequently used in the literature. The main benefits of the pHLM assay are as follows: it is relatively fast and easy to perform, it is not labor intensive, and it can be applicable for the prediction of phase I SCs metabolites in urine and blood samples (13–15).

4F-MDMB-BICA, as the newest dangerous illicit SC, was identified in 51 different police seizures and 11 deaths were attributed to its consumption in Hungary until 11 August 2020 (16). Chemically, it possesses an indole core, a 4-fluorobutyl tail, a carboxamide linker group and a methyl dimethylbutanoate non-cyclic linked group. It shows close structural similarity to 5F-methyl-2-[[1-(5-fluoropentyl)indole-3-carbonyl]amino]-3,3-dimethyl-butanoate (5F-MDMB-PICA), which has one more methylene group in the fluoroalkyl tail. The aim of the present work is to identify urine and blood marker phase I metabolites of 4F-MDMB-BICA by investigating its metabolism using ultra-high performance liquid chromatography coupled to quadrupole-Orbitrap high-resolution mass spectrometry (UHPLC–HRMS-MS). The tentatively identified *in vitro* metabolites were compared in qualitative and semi-quantitative manners to *in vivo* metabolites of urine and blood samples collected from suspected drug users. This study provides the first characterization of the *in vitro* and *in vivo* metabolites of 4F-MDMB-BICA with related reliable biomarkers for analyses. The UHPLC coupled with triple-quadrupole tandem low-resolution mass spectrometry (UHPLC–MS-MS), our routine analytical method for screening blood and urine SCs, was further developed and successfully applied by adding selected characteristic metabolites and parent compound 4F-MDMB-BICA.

## Materials and Methods

### Chemicals and standards

The solution of 4F-MDMD-BICA (systematic name: methyl2-(1-(4-fluorobutyl)-1H-indole-3-carboxamido)-3,3-dimethylbutanoate; alternative abbreviations: 4F-MDMB-BUTICA, 4F-MDMB-2201) was kindly provided by the Drug Investigation Department of the Hungarian Institute for Forensic Sciences. The reference standard solution (1 mg/mL in methanol) for 4F-MDMB-BICA was originally obtained by methanolic extraction of the compound from a seized sample. AB-FUBINACA-d_4_ was purchased from Cayman Chemicals (Ann Arbor, MI, USA) and used as an internal standard (IS). For the enzymatic treatment of human liver microsomes, incubation media and NADPH-regenerating system solutions were obtained from Corning^®^ Gentest™ (Tewksbury, MA, USA). β-Glucuronidase from *Helix pomatia* (Sigma Aldrich, St. Louis. MO, USA) was used for urinary enzymatic hydrolysis. All chemicals (liquid chromatography-mass spectrometry (LC–MS) grade water, methanol, acetonitrile, ethyl acetate, formic acid and high-performance liquid chromatography (HPLC) grade ammonia solution) for sample preparation and chromatographic measurement were from VWR (Radnor, PA, USA). Biochemistry grade ammonium sulfate was provided by Merck (Darmstadt, Germany).

The standard solutions, prepared in methanol, were stored at −20°C.

### pHLM assay and sample preparation procedure of urine and blood

The pHLM incubation was performed as previously detailed (5, 17) with slight modification. Briefly, samples after 30 min of treatment were extracted with ethyl acetate followed by solvent evaporation and re-solution in 200 µL mobile phase (1:1, *A*/*B*, v/v).

The control (drug-free) urine and whole blood samples were provided by volunteers. Five authentic urine and related two blood samples were collected from suspected drug abusers.

For enzymatic hydrolysis of glucuronidated phase II metabolites of 4F-MDMB-BICA, 395 µL sodium acetate buffer (pH 5), 2.7 µL β-glucuronidase and 5 µL IS (2 µg/mL in ethanol) were added to 2 mL urine and incubated at room temperature overnight. The reaction solution was alkalized by 60 µL of 5 v/v% of ammonia solution and, after the addition of 4 mL ethyl acetate, vortexed thoroughly and centrifuged at room temperature at 1,750 × g for 10 min (K 26 D, Germany). Next, 3 mL of the upper organic layer was evaporated to dryness at 50°C, the residue was dissolved in 200 µL mobile phase (1:1, *A*/*B,* v/v) and 10 µL was injected for analysis.

For the enrichment of SCs and related metabolites from blood, 10 µL of IS (2 µg/mL in ethanol), 1.2 g ammonium sulfate and 1.5 mL of acetonitrile were added to 1 mL whole blood sample and the solution was vortexed thoroughly for 1 min. In the following step, after 10 min centrifugation at 1,750 × g (K 26 D, Germany), 1 mL of the upper layer was evaporated to dryness at 50°C. The dried extract was dissolved in 200 µL mobile phase (1:1, *A*/*B*, v/v) and centrifuged (Universal 320 R, Hettich, Tuttlingen, Germany) at 21,000 × g for 10 min at room temperature. Aliquots (10 µL) of the supernatant were injected for analysis.

### UHPLC–HRMS and UHPLC–HRMS-MS conditions

The UHPLC–HRMS and UHPLC–HRMS-MS measurements were performed on Waters Acquity I-Class UPLC™ (Milford, MA, UK) coupled to Thermo Scientific Q Exactive™ Plus Hybrid Quadrupole-Orbitrap™ (Waltham, MA, USA) mass spectrometer.

Chromatographic separation was carried out on Accucore™ C30 column (150 × 2.1 mm, 2.6 µm) with an equivalent guard column (10 × 2.1 mm, 2.6 µm) from Thermo Fisher Scientific (Waltham, MA, USA). The UHPLC mobile phase *A* consisted of 0.1% formic acid solution, and mobile phase *B* was composed of acetonitrile with 0.1%  v/v formic acid. The gradient programme started with 0.4 mL/min flow rate and 20% *B*, ramped to 60% B in 15 min, then ramped to 100% *B* and 0.7 mL/min flow rate in 0.5 min, held for 2 min, returned to 20% *B* in 0.5 min, held for 1.5 min, returned to 0.4 mL/min flow rate in 0.1 min, and the initial condition held for 0.4 min. Column temperature was maintained at 50°C, the autosampler temperature was 15°C and 10 µL samples were used for analysis.

The mass spectrometer was operated in positive electrospray ionization (ESI) mode. The instrument settings were as follows: capillary temperature 262.5°C, S-Lens radio frequency (RF) level 50, spray voltage 3.5 kV, sheath gas flow 50, spare gas flow 2.5 and auxiliary gas flow 12.50 in arbitrary unit. The full-scan maximum injection (IT) time was 100 ms for a mass range from 150 to 600 *m/z* with a resolution of 70,000 (full width at half maximum (FWHM)), and the automatic gain control (AGC) setting was defined as 3 × 10^6^ charges. In parallel reaction monitoring (PRM) with resolution of 17,500 (FWHM), the AGC setting was defined as 3 × 10^6^ charges and the maximum IT was set to 30 ms. The width of the isolation window of precursor ion was 1.0 Da. The collision energy was stepped, with variable energies at 20, 30, and 45 eV.

MassLynx 4.1 (Milford, MA, USA) software was used for UPLC, and Xcalibur 4.3.73.11 software (Waltham, MA, USA) was used for data, chromatograms and spectra acquisitions.

### UHPLC–MS-MS parameters

Chromatographic separations were performed with Shimadzu Nexera (Kyoto, Japan) UHPLC system using Kinetex C18 column (100 × 2.1 mm, 2.6 µm) with an equivalent guard column (4 × 2 mm, 5 µm) from Phenomenex (Torrance, CA, USA). The column temperature was set to 50°C. The mobile phase was identical with that used in UHPLC--HRMS-MS. Total run time was 10 min and the following gradient programme was used for separation: started with 0.4 mL/min flow rate and 40% *B* ramped to 80% *B* in 5 min, then ramped to 100% *B* in 1 min, held for 0.5 min, then ramped to 0.9 mL/min flow rate in 0.1 min, held for 0.8 min, returned to 40% *B* within 0.1 min, held for 2 min, then returned to 0.4 mL/min flow rate within 0.1 min, and held the initial condition for 0.4 min. Injection volume of 15 µL was used.

The TSQ Fortis triple quadrupole mass spectrometer (Thermo Scientific, Waltham, MA, USA) was operated in positive ESI mode. The instrument settings were as follows: capillary temperature 300°C, vaporizer temperature 350°C, spray voltage 4.5 kV, sheath gas flow 50, sweep gas flow 1 and auxiliary gas flow 5 in arbitrary units. Data acquisition was performed in the selected reaction monitoring mode with both Q1 and Q3 resolution FWHM at 0.7 and collision-induced dissociation (CID) gas at a pressure of 1.5 mTorr in the collision cell. The flow injection method was performed in order to optimize the ESI source and determine the proper quantifier and qualifier ions of given protonated precursor ions and optimize the collision energy and tube lens for each analyte.

The UHPLC was controlled using the LabSolution software (Shimadzu, Kyoto, Japan). Data were acquired with Xcalibur 4.2.28.14 software (Thermo Fisher Scientific, Waltham, MA, USA).

### Data processing

The UHPLC–HRMS raw files of *in vitro* samples (control pHLM, 4F-MDMB-BICA treated by pHLM) were imported into the Progenesis QI (Nonlinear Dynamics, Newcastle, United Kingdom) software for the alignment of retention times, peak selection (in more sensitive mode), deconvolution and determination of peak intensities of molecular ions. Multiple adduct ions ([M + H]^+^, [M + Na]^+^, [M + K]^+^, [M + ACN + H]^+^ and [M-H_2_O + H]^+^) were selected to identify and remove redundant adduct ion forms from all detected ions. The obtained data matrix, containing retention times, accurate masses and peak intensities was evaluated statistically by analysis of variance (ANOVA) integrated in Progenesis QI. The tentative metabolites of 4F-MDMB-BICA were statistically identified by using criteria such as ANOVA (*P* value) ≤0.05 and max fold change ≥20 for compounds of treated samples.

The UHPLC–MS-MS raw files were processed manually in order to obtain qualitative information. For quantitative evaluation, the integrated processing setup of Xcalibur software was used.

### UHPLC–MS-MS method validation

The UHPLC–MS-MS method was validated in urine and blood using five replicates per level. The limit of detection (LOD), limit of quantification (LOQ) and linearity were determined by spiking different concentrations of 4F-MDMB-BICA into drug-free urine (0, 0.02, 0.2, 1, 2 and 5 ng/mL) and blood (0, 0.04, 0.4, 2, 4 and 10 ng/mL) in three times. LOD and LOQ values were calculated, respectively, on the basis of the standard deviation (SD) and the slope (S) of the calibration curve.

$$\begin{eqnarray*}
{\rm{LOD}} \!\!\!&=&\!\!\! {{3.3{\ } \times {\rm{ SD}}} \over {\rm{S}}}\\
{\rm{LOQ}} \!\!\!&=&\!\!\! {{10{\ } \times {\rm{ SD}}} \over {\rm{S}}}\vspace*{-6pt}\end{eqnarray*}$$

Accuracy and precision were determined by analyzing samples at “low” (0.02 ng/mL in urine and 0.4 ng/mL in blood), “mid” (1 ng/mL in urine and 2 ng/mL in blood) and “high” (5 ng/mL in urine and 4 ng/mL in blood) concentrations in three analytical runs.

Carry-over was assessed by injecting a drug-free urine sample after injecting a high concentration of analyte (5 ng/mL in urine and 10 ng/mL in blood). To evaluate the stability of 4F-MDMB-BICA, “low”, “mid” and “high” samples were re-injected 24 hr after the first injection and compered to the original concentrations. Results of the second runs were expressed as the percentage of their respective values in the first run.

The recovery, matrix effect and process efficiency were determined in accordance with the procedure of Matuszewski et al. and Cappiello et al. (18, 19) at “low” (0.02 ng/mL in urine and 0.4 ng/mL in blood), “mid” (1 ng/mL in urine and 2 ng/mL in blood) and “high” (5 ng/mL in urine and 4 ng/mL in blood) concentrations.

## Results and Discussion

### UHPLC–HRMS-MS analysis of 4F-MDMB-BICA

**Figure 1.**
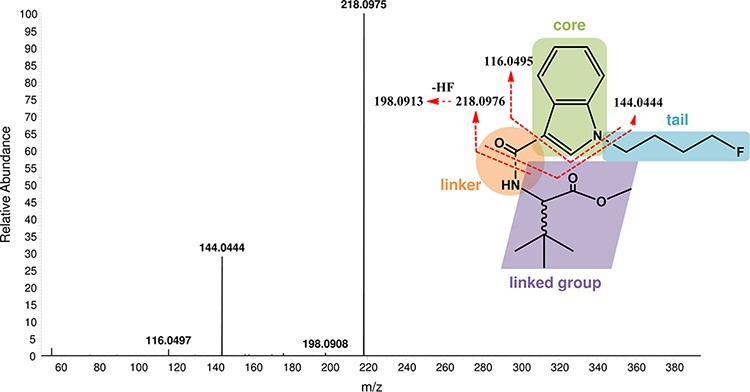
Structure of 4F-MDMB-BICA with labeling of main structure part (green, core; blue, tail; orange, linker; purple, linked group), proposed fragmentations (theoretical mass) and their HRMS-MS spectra with observed *m*/*z* values.

For UHPLC–HRMS-MS analysis of 4F-MDMB-BICA, the appropriate transitions (quantifier and qualifier ions) were selected from HRMS-MS scans and related CEs with ESI parameters were optimized (Figure 1). The investigation of the ESI-HRMS-MS behavior of single-charged protonated 4F-MDMB-BICA molecular ion [M + H^+^] at 363.2085 *m/z* (theoretical mass of 363.2078 *m/z*) resulted in the production of two main fragment ions at 218.0975 *m/z* (theoretical mass of 218.0976 *m/z*) and 144.0444 *m/z* (theoretical mass of 144.0444 *m/z*) as the quantifier and qualifier ions, respectively. The most abundant quantifier ion was produced by losing the methyldimethylbutanoate moiety linked to the carboxamide group via cleavage of the amide bond. An additional neutral loss of the 4-fluorobutyl tail group from the indole core formed the qualifier ion (Figure 1). The retention time for 4F-MDMB-BICA was found to be 11.54 min.

### *In vitro* metabolic profiling of 4F-MDMB-BICA

For the characterization of *in vitro* and *in vivo* metabolism of 4F-MDMB-BICA, non-targeted UHPLC–HRMS and semi-targeted UHPLC–HRMS-MS methods were developed with related data evaluation. In order to obtain high-efficiency UHPLC reversed-phase separation of metabolites, in particular, considering isomers, a relatively long (150 mm) C30 column with core–shell particles was used. After 30 min pHLM incubation of 4F-MDMB-BICA, 30 metabolites were tentatively identified by their fragmentation pathways. In the first step of selection of putative metabolites of 4F-MDMB-BICA, three repeated UHPLC–HRMS analyses, each for control pHLM and 4F-MDMB-BICA-treated pHLM samples, were performed. For the preparation of peak list and selection of accurate mass/charge values of molecular ions with related retention times of candidate metabolites, the resulting HRMS raw files were processed. The obtained HRMS data of control and treated pHLM samples were statistically compared by Progenesis QI software, and then the putative metabolites were manually filtered based on literature data (20–23). Molecular ions selected manually were further studied by semi-targeted UHPLC–HRMS-MS measurement in order to obtain structural information of interests. The possible substructures or structures of unknown molecular ions were determined by manually comparing the related HRMS-MS spectra with their hypothetical fragmentation, which was based on fragmentation of the parent compound and literature data of other SCs with similar core, linker and linked group (20–23). Metabolites and parent compound identified tentatively were ranked according to decreasing mean peak area ratio of quantifier ions of given substance and AB-FUBINACA-d_4_ using three replicate PRM measurements. The highest rank was given to the compound with the highest area ratio. However, it is important to note that the obtained ranking is informative and does not reflect the exact quantity order, because not all of the metabolite standards, essential for the development of their accurate quantitation, are not available.

**Figure 2.**
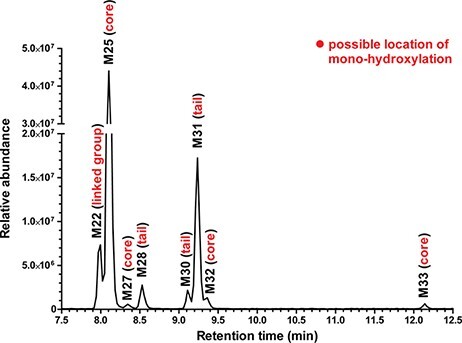
UHPLC–HRMS-MS extracted ion chromatogram of mono-hydroxylation isomers labeled with the assumed location of hydroxylation obtained from pHLM sample.

**Figure 3.**
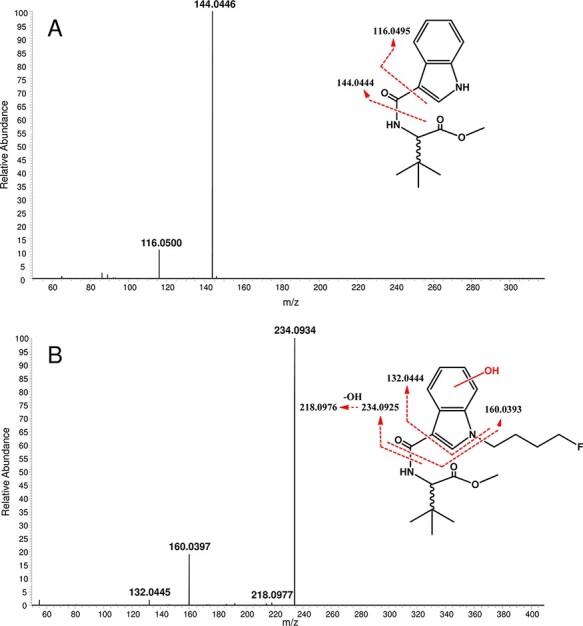
HRMS-MS spectra of 4F-MDMB-BICA metabolites M18 (A) and M25 (B) with observed m/z values, and their proposed substructures and related fragmentations (with theoretical mass) obtained from pHLM samples.

The screening of 4F-MDMB-BICA metabolites in pHLM samples with our UHPLC–HRMS-MS method gave a relatively high number of 30 metabolites compared with the number of metabolites of 5F-MDMB-PICA reported previously (20, 21). The application of C30 column allowed us to separate the isomers of given metabolites such as those of ester hydrolysis + mono-hydroxylation within 20 min of total run time. The extracted ion chromatograms of mono-hydroxylation isomers are shown in Figure 2. Although several literature methods related to the separation of SCs have been described the application of ammonium formate as eluent modifier in different concentrations, we did not find any benefit of its use (7, 12–15, 17, 21). Furthermore, based on our results, an ion suppression effect was attributed to this compound. Therefore, only formic acid was used as eluent additive during chromatographic separation. The main UHPLC–HRMS-MS parameters and tentatively identified metabolites with structural information are summarized in Table I. The pHLM assays provided several biotransformations of the parent compound involving its core, tail, linker and linked moieties such as ester hydrolysis, hydroxylations, dealkylation, oxidative defluorination and dehydrogenation, as well as their combinations. Considering the rank sequence, the highest area ratios were observed in the case of *N*-dealkylated (M18) and mono-hydroxylated (M25) products (Table I). The fragmentation pattern of M18 is illustrated in Figure 3a. In interpretation of the HRMS-MS spectrum of metabolite **M25**, the position of hydroxylation was located on the indole core (Figure 3b). Swortwood and co-workers reported that the ester-hydrolyzed + oxidative defluorinated form of 5F-MDMB-PICA was found to be the main metabolite in human hepatocyte incubation (21). Interestingly, this kind of biotrasformation was not identified in pHLM assay. However, the presence of ester hydrolysis + dehydrogenation metabolite (M26) with a 0.02 Da difference from the ester-hydrolyzed + oxidative defluorinated metabolite was confirmed with both UHPLC–HRMS and UHPLC–HRMS-MS measurements in pHLM and biofluids (Table I).

**Table I.** The Main UHPLC--MS, UHPLC--MS-MS and Structural Information of Identified Phase I Metabolites of 4F-MDMB-BICA P in Order of Their RT

ID	Biotransformation	Characteristic main metabolite in	Assumed location of biotransformation	General formula	Theoretical mass of [M + H]^+^ (*m*/*z*)	Measurement error [ppm]	RT [min]	Theoretical mass of fragment ions (*m*/*z*)	Ranking							
									pHLM	Urine 1	Urine 2	Urine 3	Urine 4	Urine 5	Blood 1	Blood 2

P	4F-MDMB-BICA			C_20_H_27_FN_2_O_3_	363.2078	2.1	11.54	218.0976 144.0444	1	ND	17	ND	ND	ND	1	2
M01	Ester hydrolysis +*N-*dealkylation +dehydrogenation		Linked group and core and linked group	C_15_H_16_N_2_O_3_	273.1234	2.3	3.75	144.0444 116.0495	13	6	3	ND	ND	ND	ND	7
M02	*N-*dealkylation +mono-hydroxylation		Core and linked group	C_16_H_20_N_2_O_4_	305.1496	1.4	3.90	144.0444 162.1125	20	ND	13	ND	ND	ND	ND	ND
M03	*N-*dealkylation +mono-hydroxylation		Core and core	C_16_H_20_N_2_O_4_	305.1496	1.6	4.02	160.0393 132.0444	17	ND	18	ND	ND	ND	ND	ND
M04	*N-*dealkylation +mono-hydroxylation		Core and core	C_16_H_20_N_2_O_4_	305.1496	2.0	4.55	160.0393 132.0444	8	9	8	ND	ND	ND	ND	ND
M05	Di-hydroxylation		Core and linked group	C_20_H_27_FN_2_O_5_	395.1977	0.8	4.72	234.0925 160.0393	25	ND	ND	ND	ND	ND	ND	ND
M06	Oxidative defluorination +mono-hydroxylation		Tail and core	C_20_H_28_N_2_O_5_	377.2071	4.0	5.34	232.0968 160.0393	30	13–14	15	ND	ND	ND	ND	ND
M07	*N-*dealkylation +mono-hydroxylation		Core and core	C_16_H_20_N_2_O_4_	305.1496	1.7	5.61	160.0393 132.0444	21	12	11	ND	ND	ND	ND	ND
M08	Ester hydrolysis +mono-hydroxylation		Linked group and core	C_19_H_25_FN_2_O_4_	365.1871	−0.3	5.65	234.0925 160.0393	26	3	4	ND	3	ND	ND	9
M09	Ester hydrolysis +oxidative defluorination		Linked group and tail	C_19_H_26_N_2_O_4_	347.1965	3.5	5.68	216.1019 144.0444	ND	ND	14	ND	ND	ND	ND	12–13
M10	Di-hydroxylation		Tail and tail	C_20_H_27_FN_2_O_5_	395.1977	0.8	5.71	144.0444 250.0874	27	ND	ND	ND	ND	ND	ND	ND
M11	Ester hydrolysis +mono-hydroxylation		Core + linked group	C_19_H_25_FN_2_O_4_	365.1871	−1.4	5.90	218.0976 144.0444	28	5	6	ND	ND	ND	ND	5
M12	Amide hydrolysis		Linker group	C_13_H_14_FNO_2_	236.1081	−4.1	6.00	118.0651 130.0651	12	7	10	ND	ND	2	ND	ND
M13	Ester hydrolysis +mono-hydroxylation		Linked group and tail	C_19_H_25_FN_2_O_4_	365.1871	−1.1	6.07	234.0925 144.0444	ND	10	ND	ND	ND	ND	ND	ND
M14	Di-hydroxylation		Core and tail	C_20_H_27_FN_2_O_5_	395.1977	0.8	6.13	160.0393 250.0874	29	ND	ND	ND	ND	ND	ND	ND
M15	Di-hydroxylation		Core and tail	C_20_H_27_FN_2_O_5_	395.1977	1.1	6.50	160.0393 250.0874	19	ND	ND	ND	ND	ND	ND	ND
M16	Ester hydrolysis +mono-hydroxylation		Linked group and core	C_19_H_25_FN_2_O_4_	365.1871	0.3	6.70	234.0925 144.0444	31	15	20	ND	ND	ND	ND	ND
M17	Di-hydroxylation		Tail and tail	C_20_H_27_FN_2_O_5_	395.1977	0.8	6.90	335.1765 250.0874	18	ND	ND	ND	ND	ND	ND	ND
M18	*N-*dealkylation		Core	C_16_H_20_N_2_O_3_	289.1547	2.2	7.32	144.0444 116.0495	2	ND	19	ND	ND	ND	ND	3
M19	Carboxylation		Linked group	C_20_H_25_FN_2_O_5_	393.1820	1.2	7.41	218.0976 144.0444	24	13–14	9	ND	ND	ND	ND	ND
M20	*N-*dealkylation +mono-hydroxylation		Core and core	C_16_H_20_N_2_O_4_	305.1496	1.3	7.43	160.0393 132.0444	23	ND	ND	ND	ND	ND	ND	ND
M21	Butanoic acid		Tail	C_10_H_26_N_2_O_5_	375.1914	−2.1	7.97	230.0812 144.0444	ND	4	7	ND	ND	ND	ND	8
M22	Mono-hydroxylation		Linked group	C_20_H_27_FN_2_O_4_	379.2028	2.6	7.98	218.0976 144.0444	7	ND	12	ND	ND	ND	ND	ND
M23	Di-hydroxylation		Tail and tail	C_20_H_27_FN_2_O_5_	395.1977	1.5	8.09	335.1765 250.0874	22	ND	ND	ND	ND	ND	ND	ND
M24	Oxidative defluorination		Tail	C_20_H_28_N_2_O_4_	361.2122	3.1	8.09	216.1019 144.0444	16	ND	ND	ND	ND	ND	ND	6
M25	Mono-hydroxylation	pHLM (1st), Urine (2nd)	Core	C_20_H_27_FN_2_O_4_	379.2028	2.7	8.09	234.0925 160.0393	3	2	2	1	1	3	ND	10
M26	Ester hydrolysis +dehydrogenation	pHLM (2nd), Blood (2nd)	Linked group and linked group	C_19_H_23_FN_2_O_3_	347.1765	3.0	8.17	218.0976 144.0444	4	8	5	ND	ND	ND	3	4
M27	Mono-hydroxylation		Core	C_20_H_27_FN_2_O_4_	379.2028	1.1	8.34	234.0925 144.0444	15	ND	ND	ND	ND	ND	ND	12–13
M28	Mono-hydroxylation		Tail	C_20_H_27_FN_2_O_4_	379.2028	2.4	8.53	234.0925 144.0444	9	ND	ND	ND	ND	ND	ND	14
M29	Ester hydrolysis	Urine (1st), Blood (1st)	Linked group	C_19_H_25_FN_2_O_3_	349.1922	2.3	8.83	218.0976 144.0444	6	1	1	2	2	1	2	1
M30	Mono-hydroxylation		Tail	C_20_H_27_FN_2_O_4_	379.2028	1.8	9.11	144.0444 234.0925	11	ND	ND	ND	ND	ND	ND	ND
M31	Mono-hydroxylation		Tail	C_20_H_27_FN_2_O_4_	379.2028	2.5	9.24	144.0444 234.0925	5	ND	ND	ND	ND	ND	ND	ND
M32	Mono-hydroxylation		Core	C_20_H_27_FN_2_O_4_	379.2028	2.4	9.36	234.0925 160.0393	10	11	16	ND	ND	ND	ND	ND
M33	Mono-hydroxylation		Core	C_20_H_27_FN_2_O_4_	379.2028	1.9	12.11	234.0925 160.0393	14	ND	ND	ND	ND	ND	ND	11

For ranking, the mean peak area ratios of each metabolite and IS were compared. Quantifier (upper) and qualifier (lower) fragment ions were obtained by the UHPLC–MS-MS method.

ND, not detected; P, parent compound; RT, retention times.

### *In vivo* metabolic profiling of 4F-MDMB-BICA

For determination of putative *in vivo* metabolites of 4F-MDMB-BICA, five authentic urine samples were screened by the semi-targeted UHPLC–HRMS-MS method (precursor ions of metabolites obtained by pHLM assay using full retention time window in PRM including list) with the possibility of identification of new metabolites.

**Figure 4.**
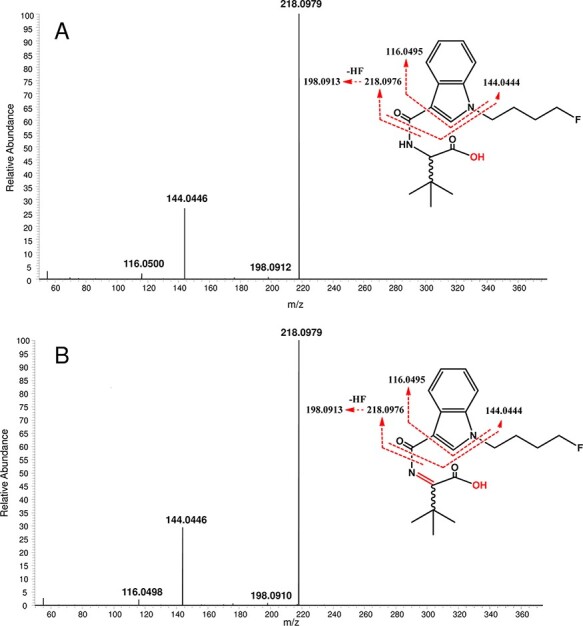
HRMS-MS spectra of M29 (A) and M26 (B) with their proposed substructures with related fragmentations (with theoretical mass) obtained from pHLM samples.

Overall, 20 metabolites were tentatively identified in authentic urine samples and provided three new metabolites, namely ester-hydrolyzed + oxidative defluorination (M09), ester hydrolysis + mono-hydroxylation (M13) and butanoic acid metabolite (M21) compared with metabolites obtained by pHLM assay. However, not all isomers of di-hydroxylation (M05, M10, M14, M15, M17 and M23), *N*-alkylation + mono-hydroxylation (M20), oxidative defluorination (M24) and mono-hydroxylation (M27, M28, M30, M31 and M33) were detected in urine samples (Table I). Specifically, mono-hydroxylated metabolites (M28, M30 and M31) bearing the hydroxyl group on the butyl tail were not detected in urine and blood samples. By ranking the area ratio of urine metabolites, similar to MDMB-4en-PINACA, 5F-MDMB-PICA, 5F‐AMB, 4F-MDMB-BINACA and AMB-FUBINACA, the M29 ester hydrolysis substance was identified as the main metabolite (Table I) (20–26). The obtained HRMS-MS spectrum of M29 is shown in Figure 4a. In terms of predicting main urine metabolites between pHLM and human liver hepatocyte assays, the M29 of 4F-MDMB-BICA was only the fifth most abundant in pHLM samples in our case. In comparison, the ester hydrolysis product of 5F-MDMB-PICA was determined with the highest rank in the human liver hepatocyte samples by Swortwood et al. (21). Interestingly, for non-hydrolytic metabolites, the phenomenon was completely the opposite. For instance, mono-hydroxylation metabolites were presented in very low amounts in the hepatocyte samples (21, 24). The mono-hydroxylation isomer (M25) with modification on the indole core appeared as the second most abundant metabolomics product in urine.

The mono-hydroxylation isomer (M25) with modification on the indole core appeared as the second most abundant metabolomics product in urine. Unfortunately, there was no information available for the quantity of administered 4F-MDMB-BICA and time passed between the use and sample collection. Therefore, it is difficult to give a reliable explanation for the large difference in the number of detected metabolites in urine samples such as Urine 1 (15), Urine 2 (19), Urine 3 (2) Urine 4 (3) and Urine 5 (3). Interestingly, the parent compound 4F-MDMB-BICA was detected only in Urine 2 sample. The consumption of this new illicit SC could be confirmed only by the detection of metabolites in the other four cases. This example demonstrates the effectiveness of the metabolomics approach of SCs analysis in toxicology and forensic science and practice.

Additional *in vivo* metabolomics details of 4F-MDMB-BICA was obtained by profiling two authentic blood samples. The Urine 1-Blood 1 and Urine 2-Blood 2 samples are paired collections from the same individuals. The semi-targeted UHPLC–HRMS-MS measurement of the blood samples did not result in any additional new metabolite. Altogether, 13 metabolites were associated with 4F-MDMB-BICA. Similar to Urine 2 sample, the highest number of metabolites were found in Blood 2 sample (11), while only the ester hydrolysis and ester hydrolysis + dehydrogenated substances, selected as main metabolites, were detectable in Blood 1. The second highest rank was assigned to *N*-dealkylation (M18) substance, but it cannot be selected as a characteristic biomarker of exogenous 4F-MDMB-BICA. The reason is that it is a common metabolite with 5F-MDMB-PICA, just like the ester hydrolysis + *N*-dealkylation + dehydrogenation (M01) and *N*-dealkylation + mono-hydroxylation isomers (M02, M03, M04, M07 and M20) (20, 21). The hydrolysis + dehydrogenation (M26) metabolites were detected in both blood samples. Therefore, M26 metabolites were proposed as additional blood biomarkes of 4F-MDMB-BICA (Figure 4b). In contrasts to urine samples, the parent compound had the highest rank in both blood samples.

### Validation and application of routine UHPLC–MS-MS method for quantitation of 4F-MDMB-BICA in urine and blood samples, and qualitative screening of selected characteristic metabolites

**Figure 5.**
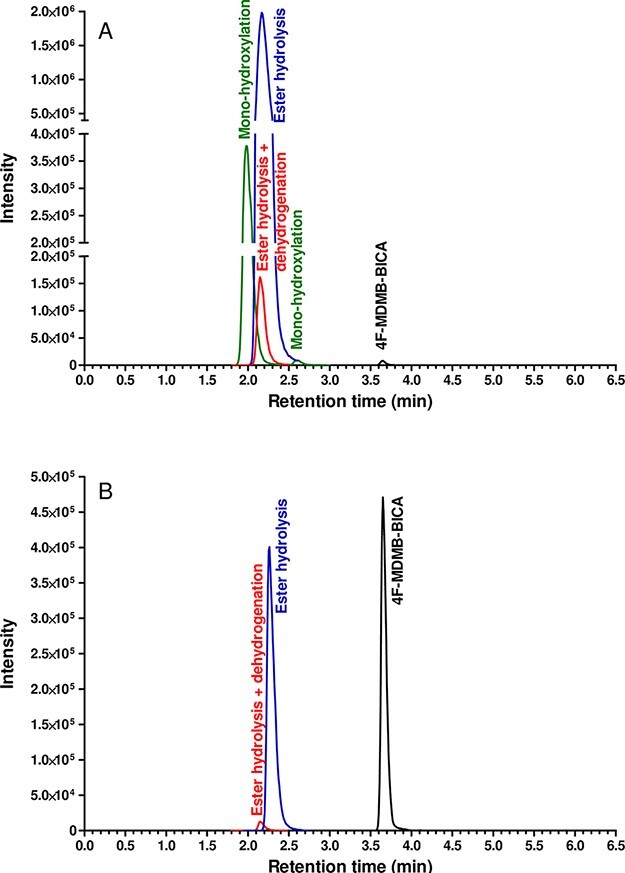
Extracted ion chromatogram of 4F-MDMB-BICA, ester hydrolysis, mono-hydroxylation, ester hydrolysis + mono-hydroxylation and ester hydrolysis + dehydrogenation obtained by targeted UHPLC–MS-MS analysis of urine 2 (a) and blood 2 (b) sample.

For forensic purposes, the obtained UHPLC–HRMS-MS metabolomics results provided reliable information for updating our UHPLC–MS-MS method for quantitative analysis of urine 4F-MDMB-BICA and the qualitative screening of M29 primary metabolite, and M25 (for urine) and M26 (for blood) secondary metabolites. The routine chromatographic analysis was performed on the shorter C18 core-shell column, under fast gradient elution within 6.5 min analysis time, leading to less efficient separation for isomers. Thus, for selected transitions, the given biotransformations were indicated instead of isomer names. The high-resolution analytical method was successfully converted to the UHPLC–MS-MS method by monitoring two transitions per parent compound and two metabolites within 6.5 min analysis time (Table II). The method was validated for 4F-MDMB-BICA quantification in human urine and blood by investigating the LOD, LOQ, linearity, accuracy, precision, carry-over, recovery, matrix effect and process efficiency. For urine and blood samples, the main validation criteria and obtained validation parameters of 4F-MDMB-BICA are summarized in Table S1. For 4F-MDMB-BICA, our UHPLC–MS-MS method provided 0.003 ng/mL of LOD in urine and 0.004 ng/mL in whole blood sample. Concerning LOQ values, 0.010 ng/mL urine and 0.011 ng/mL blood for 4F-MDMB-BICA were obtained. For matrix effect, recovery and process efficiency, the obtained parameters fulfilled the validation criteria except for matrix effect at low concentration samples. The recovery of 4F-MDMB-BICA was determined at low, mid and high concentration levels, and it was between 74 and 119% in urine and between 74 and 129% in blood. The calibration curves for the parent compound were linear (R2 ≥ 99%) in the range of 0.02–5 ng/mL urine and 0.04–10 ng/g blood. Accuracy of targeted UHPLC–MS-MS in three different concentration levels was found within an acceptable interval of 5% in urine and blood samples. As shown in Table S1, the obtained precision ranged from 0.06 to 0.08 percent coefficient of variation (%CV) in urine and from 2.7 to 6.5 %CV in blood. The calculated carry-over of 4F-MDMB-BICA was negligible (<0.02%) in urine and blood. Overall, the obtained main validation parameters verify that our targeted UHPLC–MS-MS method with related sample preparation procedures is suitable to screen 4F-MDMD-BICA in both urine and whole blood samples (Table S1). By the application of the targeted method, the parent compound was detectable only in Urine 2 (0.970 ng/mL), Blood 1 (0.920 ng/mL) and Blood 2 (>10 ng/mL) samples. Concerning primary and secondary biomarkers, the obtained results of authentic urine and blood samples are summarized in Table III. The related extracted ion chromatograms of the Urine 2 and Blood 2 samples are illustrated in Figure 5.

**Table II.** Main UHPLC–MS-MS Parameters of 4F-MDMB-BICA, Ester Hydrolysis, Mono-Hydroxylation, Ester Hydrolysis + Dehydrogenation Metabolites and AB-FUBINACA-d_4_ (Internal Standard)

Compound	Retention time (min)	Precursor ion (m/z)	Product ion 1 (quantifier ion, m/z)	CE (eV)	Product ion 2 (qualifier ion, m/z)	CE (eV)
AB-FUBINACA-d_4_	2.19	373.2	257.1	25	–	–
Ester hydrolysis	2.33	349.2	218.1	20	144	35
Mono-hydroxylation	2.04	379.2	234.1	20	144	35
Ester hydrolysis + dehydrogenation	2.14	347.2	218.1	20	144	35
4F-MDMB-BICA	3.69	363.2	218.1	19	144	38

The tube lens was held at 95 V.

CE, collision energy.

**Table III.** Qualitative and Quantitative Results of 4F-MDMB-BICA, Ester Hydrolysis, Mono-Hydroxylation and Ester Hydrolysis + Dehydrogenation Metabolites Obtained by the UHPLC–MS-MS Method in Authentic Urine and Blood Samples

Compound	Suggested in	Urine 1	Urine 2	Urine 3	Urine 4	Urine 5	Blood 1	Blood 2
Ester hydrolysis	Urine/Blood	Detected	Detected	Detected	Detected	Detected	Detected	Detected
Mono-hydroxylation	Urine	Detected	Detected	Detected	Detected	Detected	ND	Detected
Ester hydrolysis + dehydrogenation	Blood	Detected	Detected	ND	ND	ND	Detected	Detected
4F-MDMB-BICA	Urine/Blood	ND	0.970 ng/mL	ND	ND	ND	0.920 ng/mL	>10 ng/mL

ND, Not detected.

## Conclusion

To the best of our knowledge, this is the first attempt to describe and analyze the phase I metabolites of 4F-MDMD-BICA after treatment with pHLM and in human urine and blood samples with non-targeted (UHPLC–HRMS), semi-targeted (UHPLC–HRMS-MS) and targeted (UHPLC–MS-MS) analytical methods. The selection of appropriate biomarkers of 4F-MDMB-BICA should be carried out cautiously. For instance, 5F-MDMD-PICA, currently the most widespread SC in Hungary, has common metabolites with 4F-MDMD-BICA, such as *N*-dealkylation, ester hydrolysis + *N*-dealkylation + dehydrogenation and *N*-dealkylation + mono-hydroxylation (20, 21). Overall, 33 metabolites were tentatively identified. Among them, the presence of 20 substances was confirmed in five authentic urine samples, and 13 metabolites were identified in two blood samples. Of several identified biotransformations on core, tail, linker and linked group of 4F-MDMD-BICA, the ester hydrolysis metabolic product was recommended as the primary biomarker both in urine and blood. The urinary mono-hydroxylation metabolite and the ester hydrolysis + dehydrogenation substance in blood were selected as secondary confirmatory targets for the screening of 4F-MDMD-BICA. The selected metabolites with parent compound of 4F-MDMD-BICA were adapted to our routine targeted UHPLC–MS-MS method, which was validated and applied successfully in the analysis of authentic urine and blood samples.

## Supplementary Material

bkab004_Supp
