# Supplementary material for: Analytical Methodologies for the Characterization and Analysis of the Parent Compound and Phase I Metabolites of 4F-MDMB-BICA in Human Microsome, Urine and Blood Samples
Source: J Anal Toxicol. 2021 Jan 6;46(2):135–45. doi: 10.1093/jat/bkab004 (PMC8866813; doi:10.1093/jat/bkab004)
Supplement: bkab004_Supp [file bkab004_supp.zip › jat-20-3301-File010.doc]

Supplementary information

**Analytical methodologies for the characterization and analysis of the parent compound and phase I metabolites of 4F-MDMB-BICA in human microsome, urine, and blood samples**

Tímea Körmöczi^a^, Éva Sija^b^, László Institóris^b^, Éva M Kereszty^b^, István Ilisz^a^, and Róbert Berkecz^a*^

*^a^Institute of Pharmaceutical Analysis, Faculty of Pharmacy, University of Szeged*

*Somogyi utca 4., H-6720, Szeged, Hungary*

*^b^Department of Forensic Medicine, Faculty of Medicine, University of Szeged, Kossuth Lajos sgt. 40, H-6724, Szeged, Hungary*

**^*^ Corresponding author:** Róbert Berkecz, Tel.: +36302347004, E-mail: berkecz.robert@pharm.u-szeged.hu

**Table S1.** Main validation parameters of UHPLC-MS/MS method for screening 4F-MDMB-BICA in urine and blood.

| Validation parameters | Criteria range | Urine | Blood |
| --- | --- | --- | --- |
| R2 | no criteria | 0.9937 (0.02-5 ng/mL) | 0.9902 (0.04-10 ng/mL) |
| LOD | ≤ 5 %CV | 0.003 ng/mL (2.5 %CV) | 0.004 ng/mL (0.79 %CV) |
| LOQ | ≤ 5 %CV | 0.010 ng/mL (2.5 %CV) | 0.011 ng/mL (0.79 %CV) |
| Accuracy Low (%) | 85-115 | 100.7 | 100.3 |
| Accuracy Mid (%) | 85-115 | 102.3 | 99.9 |
| Accuracy High (%) | 85-115 | 105.0 | 100.0 |
| Precision Low (%CV) | ≤ 15 | 0.6 | 3.4 |
| Precision Mid (%CV) | ≤ 15 | 0.8 | 2.7 |
| Precision High (%CV) | ≤ 15 | 0.6 | 6.5 |
| Carry-over (%) | ≤ 20 | 0.01 | 0.02 |
| Inter-day stability Low (%) | ± 15 | 3.1 | -2.4 |
| Inter-day stability Mid (%) | ± 15 | -0.2 | 2.3 |
| Inter-day stability High (%) | ± 15 | 0.6 | -1.3 |
| Recovery Low (%) | 70-130 | 118.6 | 128.8 |
| Recovery Mid (%) | 70-130 | 89.6 | 72.2 |
| Recovery High (%) | 70-130 | 74.2 | 74.2 |
| Matrix effect Low (%) | 70-130 | 62.3 | 62.0 |
| Matrix effect Mid(%) | 70-130 | 98.1 | 110.8 |
| Matrix effect High (%) | 70-130 | 121.3 | 121.5 |
| Process efficiency Low (%) | 70-130 | 73.9 | 80.1 |
| Process efficiency Mid (%) | 70-130 | 87.9 | 80.0 |
| Process efficiency High (%) | 70-130 | 89.9 | 90.2 |
| Retention stability (min) | 3.59-3.79 | 3.69 ± 0.05 | 3.69 ± 0.05 |

Coefficient of determination of linear regression (R2), limit of detection (LOD), limit of quantification (LOQ) and percent coefficient of variation (%CV).
